# Mutations in matrix and SP1 repair the packaging specificity of a Human Immunodeficiency Virus Type 1 mutant by reducing the association of Gag with spliced viral RNA

**DOI:** 10.1186/1742-4690-7-73

**Published:** 2010-09-08

**Authors:** Natalia Ristic, Mario PS Chin

**Affiliations:** 1 Aaron Diamond AIDS Research Center, The Rockefeller University, New York, New York, USA

## Abstract

**Background:**

The viral genome of HIV-1 contains several secondary structures that are important for regulating viral replication. The stem-loop 1 (SL1) sequence in the 5' untranslated region directs HIV-1 genomic RNA dimerization and packaging into the virion. Without SL1, HIV-1 cannot replicate in human T cell lines. The replication restriction phenotype in the SL1 deletion mutant appears to be multifactorial, with defects in viral RNA dimerization and packaging in producer cells as well as in reverse transcription of the viral RNA in infected cells. In this study, we sought to characterize SL1 mutant replication restrictions and provide insights into the underlying mechanisms of compensation in revertants.

**Results:**

HIV-1 lacking SL1 (NLΔSL1) did not replicate in PM-1 cells until two independent non-synonymous mutations emerged: G913A in the matrix domain (E42K) on day 18 postinfection and C1907T in the SP1 domain (P10L) on day 11 postinfection. NLΔSL1 revertants carrying either compensatory mutation showed enhanced infectivity in PM-1 cells. The SL1 revertants produced significantly more infectious particles per nanogram of p24 than did NLΔSL1. The SL1 deletion mutant packaged less HIV-1 genomic RNA and more cellular RNA, particularly signal recognition particle RNA, in the virion than the wild-type. NLΔSL1 also packaged 3- to 4-fold more spliced HIV mRNA into the virion, potentially interfering with infectious virus production. In contrast, both revertants encapsidated 2.5- to 5-fold less of these HIV-1 mRNA species. Quantitative RT-PCR analysis of RNA cross-linked with Gag in formaldehyde-fixed cells demonstrated that the compensatory mutations reduced the association between Gag and spliced HIV-1 RNA, thereby effectively preventing these RNAs from being packaged into the virion. The reduction of spliced viral RNA in the virion may have a major role in facilitating infectious virus production, thus restoring the infectivity of NLΔSL1.

**Conclusions:**

HIV-1 evolved to overcome a deletion in SL1 and restored infectivity by acquiring compensatory mutations in the N-terminal matrix or SP1 domain of Gag. These data shed light on the functions of the N-terminal matrix and SP1 domains and suggest that both regions may have a role in Gag interactions with spliced viral RNA.

## Background

HIV-1 packages two copies of the viral RNA genome, in dimeric form, through Gag-RNA interactions [1-5]. The *cis*-acting elements in the viral RNA and Gag are involved in the specific packaging of HIV-1 genomic RNA. The 5' noncoding leader sequence of the HIV-1 genome contains important *cis*-acting packaging elements. This leader region forms a series of secondary structures, including the transactivation response element, the poly(A) hairpin, the U5-PBS complex, and stem loops (SL) 1 to 4 [6-8]. Despite some sequence variations, different subtypes of HIV-1 all have similar secondary structures in this region, suggesting that the conformation of genomic RNA is important for the packaging process [9,10]. Furthermore, mutation analyses indicate that all of these structures are important for viral genomic RNA packaging [9-11]. The four SLs in the 5' untranslated region (UTR) of the viral genome act as the primary recognition sites for the nucleocapsid (NC) domain of the Gag polyprotein [7,11-16]. The NC has been shown to mediate the selection of unspliced viral genomic RNA for packaging through the interaction of its zinc finger motifs and SL3 of the viral RNA [17,18]. However, viral RNA lacking SL3 is still encapsulated into the virion [11,19], as SL1, SL2 and SL4 also interact with the NC domain during packaging [7,16].

Within the virion, HIV-1 genomic RNA exists as a dimer held together by a noncovalent linkage at the 5' end [1,4]. The dimerization process is thought to occur in the cytoplasm, and the HIV-1 genomic RNA molecules are then packaged as a dimer [3,5,20]. Though the 5' transactivation response stem-loop may play a role in HIV-1 RNA dimerization [21], the viral element that directs the dimerization process is a 6-nt palindromic sequence called the dimerization initiation signal (DIS), which is located at the loop of SL1 in the 5' UTR [3,4,9,22-26]. The DIS of two RNA molecules first form base pairs to initiate the dimerization process and form a kissing loop complex [23,24,27-29]. The NC then promotes the conversion of the kissing loop complex to a more stable extended dimer [30,31]. Recent studies have shown that base-pairing of the DIS of two RNA molecules is a major determinant in the selection of the copackaged RNA partners, and the identity of the DIS plays an important role in the copackaging of RNAs from different HIV-1 strains [3,25,32].

Given the critical role of SL1 in viral RNA dimerization and packaging, it is not surprising that deletion of SL1 from a replication competent HIV-1 molecular clone renders the virus non-infectious in human T cell lines [11,33-37]. However, SL1 deletion mutants have been shown to replicate in human PBMCs, and a primary HIV-1 isolate with a defect in RNA dimerization has been identified in a patient [35,36,38]. The underlying mechanism of this cell type-dependent restriction is unclear. Because human PBMCs are more heterogeneous in nature than T cell lines, one possibility is that a subset of the PBMC population is able to support the replication of SL1 deletion mutants. It remains to be discovered whether such a subset of cells exists or whether the presence or absence of a cellular factor is responsible for overcoming the SL1 mutant replication restriction.

Several restrictions on the replication of SL1 deletion mutant in T cell lines have been identified, including viral RNA dimerization and packaging in producer cells and reverse transcription (RT) of the viral RNA in infected cells [10,11,33-37,39,40]. Long-term culture of SL1 mutants generates revertants that retain the SL1 deletion but possess compensatory mutations in Gag [33,34,41]. SL1 deletion mutants generally package less full-length HIV-1 genomic RNA and more spliced viral RNA into the virion, whereas spliced RNA is effectively excluded from packaging in the revertants. Thus, these compensatory mutations may partially rescue SL1 deletion mutant infectivity by enhancing the packaging specificity of Gag. However, the molecular mechanism underlying the rescue of viral RNA packaging in SL1 deletion mutant revertants has not been defined. Moreover, the effects of SL1 deletion on viral RNA splicing and cellular RNA packaging are unclear.

Here we report two independent adaptations of HIV-1 that partially restored infectivity in SL1 deletion mutants in a restrictive cell line in as little as 11 days. The revertants retained the SL1 deletion but harbored compensatory mutations in Gag. SL1 deletion mutants carrying these compensatory mutations were effective in excluding spliced viral RNA from packaging. We show that reduced association between the mutated Gag and spliced viral RNA plays a major role in the exclusion of spliced HIV-1 RNAs in the revertants.

## Results and Discussion

### Replication of HIV-1 SL1 deletion mutant in PM-1 cells

**Figure 1:**
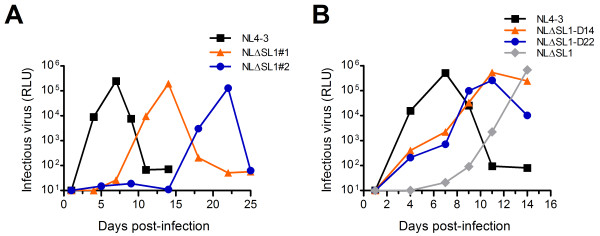
**Replication kinetics of NL4-3, NLΔSL1, and revertants**. (A) Changes in the infectivity of NLΔSL1 were observed. PM-1 cells were infected with p24-normalized NL4-3 or NLΔSL1. Virus production was measured in TZM-bl cells using culture supernatant from the infected PM-1 at different times. (B) NLΔSL1 revertants were replication competent in PM-1 cells. Culture supernatants from day 14 p.i. with NLΔSL1#1 (NLΔSL1-D14) and day 22 p.i. with NLΔSL#2 (NLΔSL1-D22) were normalized to p24 and used to infect fresh PM-1 cells, and virus production was detected as described previously.

Previous studies have shown that HIV-1 SL1 deletion mutants do not replicate in human T cell lines and that compensatory mutations that partially rescue the replication defect arise after several passages in culture [33,34,41]. In this study, a SL1 deletion mutant demonstrated delayed replication in a human T cell line. The SL1 deletion mutant, NLΔSL1, was derived from a replication-competent NL4-3 molecular clone with the 43-nt SL1 deleted (nt position 691 to 733, NL4-3 proviral DNA). In PM-1 cells infected with NLΔSL1, syncytia were observed 14 days postinfection (p.i.) in one culture and 22 days p.i. in another, whereas NL4-3 infected cells showed syncytia by 7 days p.i. (data not shown). Virus production in the infected PM-1 cells was detected in the culture supernatant 3-4 days before cytopathogenicity was observed using TZM-bl indicator cells (Figure 1A) and p24 ELISA (Additional file 1: Figure S1).

The two distinct growth kinetics of NLΔSL1 in PM-1 cells, shown in Figure 1A, suggest that variants of NLΔSL1 with enhanced infectivity may have emerged in the infected cultures on day 14 p.i. and day 22 p.i. To confirm the presence of new variants with enhanced infectivity, equal amounts of p24-normalized NL4-3, NLΔSL1 and viruses from the infected PM-1 cells on day 14 p.i. (NLΔSL1-D14) and on day 22 p.i. (NLΔSL1-D22) were used to infect fresh PM-1 cells, and virus production was monitored. NLΔSL1-D14 and NLΔSL1-D22 indeed replicated with higher efficiency than the original NLΔSL1 (Figure 1B).

### Identification of compensatory mutation in the SL1 deletion revertants

To identify the mutations responsible for the increased infectivity of NLΔSL1 and to rule out the possibility that NLΔSL1 had reverted the SL1 deletion, we isolated viral RNA from the culture supernatants, and amplified and sequenced the near full-length genome of the virus. Sequences derived from NLΔSL1-D14 and NLΔSL1-D22 showed that both variants still harbored the SL1 deletion found in NLΔSL1 (data not shown). A G913A substitution (NL4-3 numbering) was found in the matrix (MA) of NLΔSL1-D22, leading to an E42K amino acid change in the protein, and a C1907T substitution was found in the SP1 of NLΔSL1-D14, corresponding to a P10L substitution. Neither mutation had been associated with enhanced infectivity of HIV-1 prior to this study, nor did we identify additional mutations in other parts of the mutant genomes. A survey of 9675 subtype B MA protein sequences retrieved from the Los Alamos HIV Sequence Database showed that almost all sequences harbor glutamic acid at position 42, whereas lysine was detected in only 10 sequences. Leucine was not present at position 10 in any of 4454 subtype B SP1 peptide sequences retrieved from the sequence database (sequence alignments available upon request). These results suggest that these two compensatory mutations are uncommon in naturally occurring HIV-1 strains. Furthermore, these data indicate that more than one mutational pathway can compensate for the loss of SL1 secondary RNA structure.

### Compensatory mutations in *gag *rescue the replication defect of the SL1 deletion mutant

**Figure 2:**
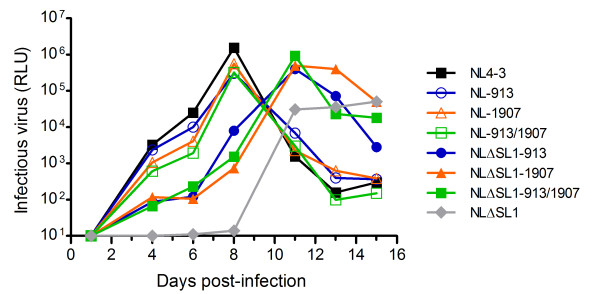
**Mutations in Gag are responsible for the changes in infectivity of NLΔSL1**. NL4-3 and NLΔSL1 carrying the G913A or C1907T mutations were normalized with p24 amount and used to infect PM-1 cells. Virus production was measured in TZM-bl cells using culture supernatant harvested from the infected cells at different times.

To verify the contribution of mutations G913A and C1907T to the enhanced infectivity of the SL1 mutant, we performed site-directed mutagenesis of NLΔSL1 to generate NLΔSL1-913, NLΔSL1-1907 and NLΔSL1-913/1907 strains. The mutant vectors were identical to the NLΔSL1 sequence, except that NLΔSL1-913 contained a G913A substitution in the MA gene, NLΔSL1-1907 had a C1907T mutation in the SP1 region and NLΔSL1-913/1907 harbored both mutations. Equal amounts of p24-normalized NL4-3, NLΔSL1, NLΔSL1-913, NLΔSL1-1907 or NLΔSL1-913/1907 were used to infect PM-1 cells, and growth kinetics were measured. SL1 deletion revertants having mutations in MA, SP1 or both demonstrated intermediate replication efficiencies between NL4-3 and NLΔSL1 (Figure 2). Combining the two mutations did not further enhance replication, as the NLΔSL1-913/1907 showed similar replication efficiency to NLΔSL1-913 and NLΔSL1-1907. This result indicates that mutation in either MA or SP1 is sufficient to partially restore the replication of the SL1 deletion mutant. NL4-3 carrying the G913A (NL-913) or C1907T (NL-1907) mutation or both (NL-913/1907) was included for comparison. None of these mutations affected the replication of the NL4-3 virus (Figure 2). Taken together, these results confirm that point mutations in MA or SP1 were responsible for the enhanced infectivity of the NLΔSL1 revertants.

### Compensatory mutations in *gag *increase the production of infectious SL1 deletion mutant virus

**Figure 3:**
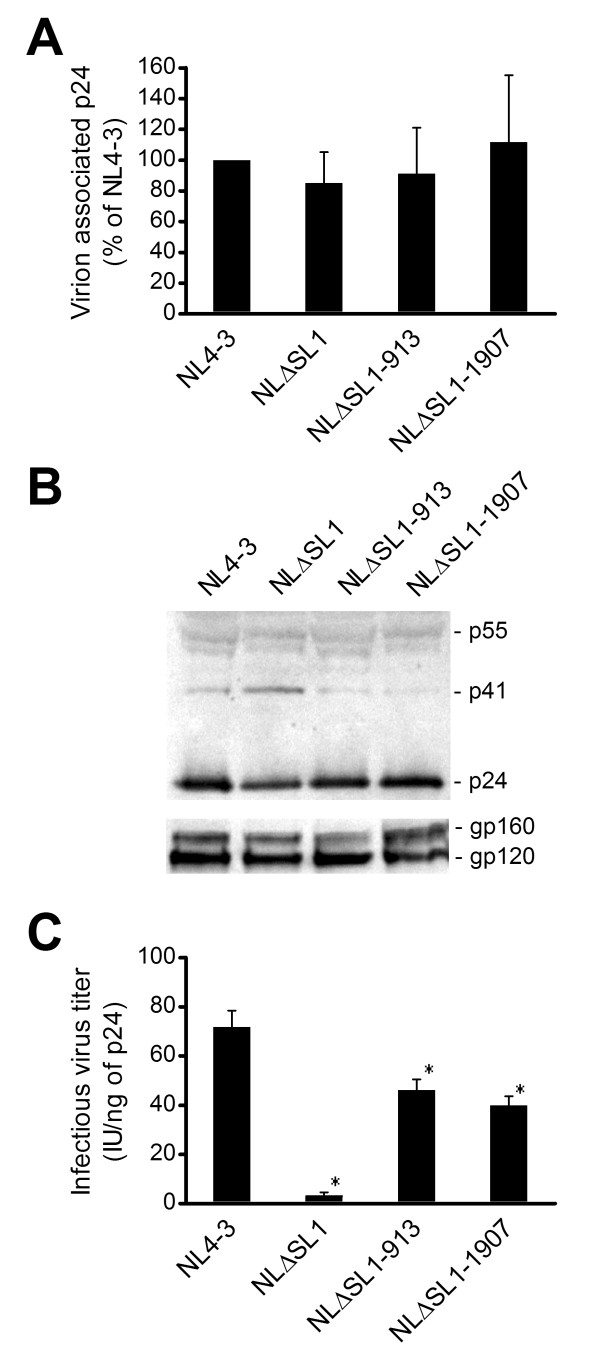
**Analyses of the production and infectivity of viral particles**. (A) Similar virus production from NL4-3 and deletion mutants. Culture supernatants of 293T cells transfected with the corresponding vectors were centrifuged through a 20% sucrose cushion. The amount of p24 in the virus pellets was determined and compared to the amount of p24 in the NL4-3 virus pellet, which was set at 100%. Means and SD of three independent experiments are shown. (B) NL4-3 and deletion mutants had similar protein expression and processing. Western blot analysis of HIV-1 virions with p24 or gp120 antiserum. The corresponding sizes of the HIV-1 proteins are shown to the right. (C) Infectious virus production varied among different mutants. Viruses harvested from the culture supernatant of 293T cells transfected with the corresponding vector were titrated for infectivity using the limiting dilution culture method in PM-1 cells. The TCID_50 _was calculated by the Reed and Muench method. The same aliquot of virus was quantified with p24 ELISA and used to normalize the titer of the virus stock. Means and SD of three independent experiments are shown. *, indicates *p *< 10^-3 ^and significant deviation from the wild-type infectious virus titer as determined by Student's *t *test.

HIV-2 carrying a mutated Ψ/SL1 reportedly has defective packaging of viral RNA and produces fewer mature particles, thus reducing the overall infectivity of the virus [42,43]. We postulated that SL1 deletion mutants could have a similar defect that affects the production of infectious virions. To evaluate virus production of the SL1 deletion mutants, we measured virion-associated p24 in the viral stocks after centrifugation through a sucrose cushion. Virus production in the deletion mutants was not affected by the absence of SL1 or by point mutations in MA or SP1 (Figure 3A). Western blot analysis of pelleted virion from cells transfected with the mutant constructs showed that the expression and processing of HIV-1 proteins were similar to those of the wild-type virus with a slight increase in unprocessed p41 (MA-capsid) in the SL1 deletion mutant (Figure 3B). However, when the same virion samples were analyzed in Western blot with p17 monoclonal antibody, we did not observe a difference in the level of MA (data not shown). We then determined the infectious titer of the virus, normalized against the amount of p24, to quantify the production of infectious virus by the mutants. NLΔSL1 produced 20-fold fewer infectious viral particles than the wild-type NL4-3 (Figure 3C), whereas NLΔSL1-913 and NLΔSL1-1907 produced only about 1.7-fold fewer infectious viruses compared to the wild type. It is likely that changes in the Gag protein sequence were responsible for the increased infectious virus production, but changes in the RNA sequence may also have played a role. We therefore investigated if the compensatory mutations in *gag *affected the infectivity of the deletion mutants at the RNA level.

### Compensatory mutations do not affect the dimerization or splicing of HIV-1 RNA

**Figure 4:**
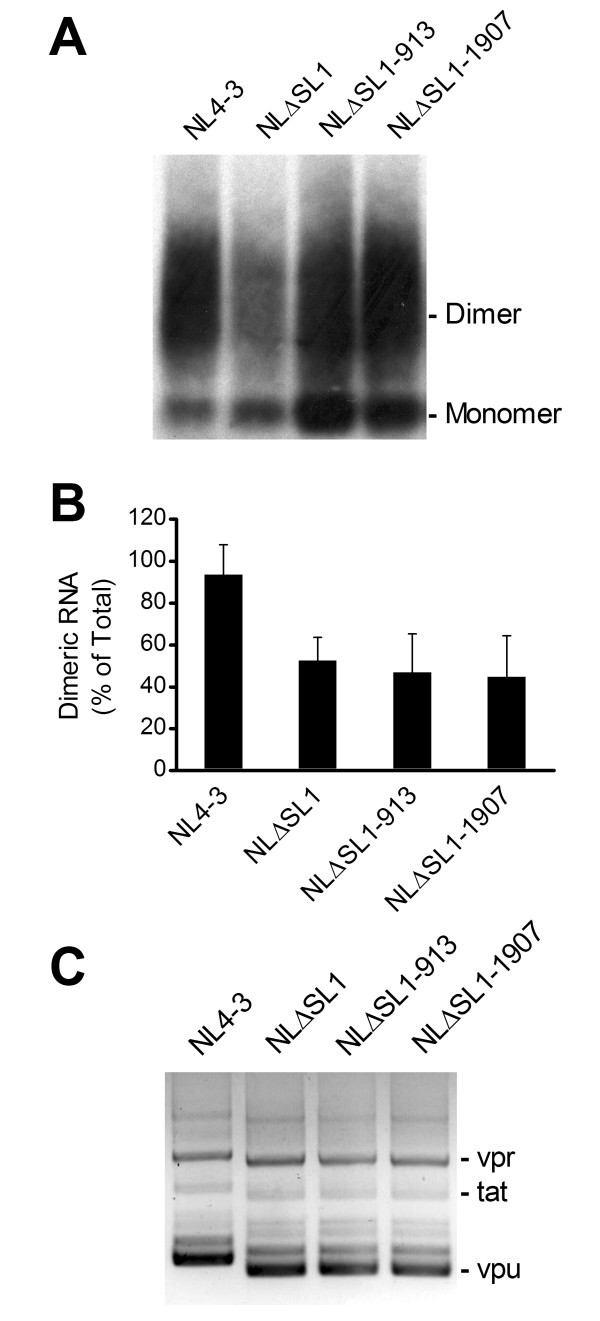
**Characterization of the dimerization state and splicing of viral RNA**. (A) Dimerization analysis of virion RNA. Virion RNAs of different proviral constructs were separated on a native agarose gel and characterized by Northern analysis. Dimer and monomer are indicated on the right side of the blot. Results are representative of two sets of experiments. (B) Compensatory mutations in gag did not affect RNA dimerization. Amounts of dimeric and monomeric RNA were quantified by densitometry, and the percentages of dimers for each construct present in the virion calculated. Means and SD of two independent experiments are shown. (C) Deletion of SL1 did not affect the splicing of HIV-1 RNAs. 293T cells were transfected with the HIV-1 constructs. Total RNA was isolated 48 hrs post-transfection and reverse-transcribed. The 4-kb singly spliced HIV-1 RNAs were amplified from cDNA and separated on an agarose gel. The SL1 deletion resulted in a population of smaller mRNAs than those observed for the wild-type HIV-1. Sequence analysis verified the identity of the products and showed that the deletion mutants had the same splicing patterns as the wild-type virus.

The SL1 of HIV-1 is responsible for directing viral RNA dimerization and is located very close to the major splice donor of the SL2 in the 5' leader sequence. We determined whether dimerization and splicing of the RNAs were affected by the deletion in SL1. Because the SL1 contains a major signal for viral RNA dimerization, we expected to find decreased levels of RNA dimer in the deletion mutant. Indeed, the NLΔSL1 had 53% dimerized RNA, compared with 94% in the wild type (Figure 4A and 4B). We then asked whether the compensatory mutations could rescue the RNA dimerization defect, and found that neither of the substitutions had a significant effect on the amount of dimeric RNA (47-45%).

We next investigated the effects of the SL1 deletion and compensatory mutations on HIV-1 RNA splicing. We specifically reverse-transcribed and amplified the 4-kb singly spliced viral RNA using primers targeting the U5 and *vpu *of the HIV-1 genome and analyzed the products by agarose gel electrophoresis. The PCR products from the wild type were as expected [44], and the identities of the bands were verified by sequencing as *vpr*, *tat *and *vpu *RNAs (Figure 4C). The SL1 deletion mutant and the revertants yielded similar products, though of smaller sizes due to the 43-nt SL1 deletion. Sequence analysis showed that the SL1 mutant and revertants used the same splicing sites as the wild type. Moreover, we did not see a marked change in RNA stability in either the wild type or the SL1 deletion mutants with the compensatory mutations (Table 1). Notably, the SL2 remains intact in the absence of SL1, confirming that RNA splicing was not affected in the NLΔSL1 mutant (Additional file 2: Figure S2). However, one has to caution that analyzing HIV-1 RNA monomer in solution may not completely reveal the elusive native structure and stability of dimeric viral RNA in the cell. Nonetheless, these data indicate that the two compensatory mutations in *gag *do not rescue infectivity in the SL1 deletion mutant by altering RNA dimerization or splicing.

**Table 1:** Stability of HIV-1 genomic RNA as predicted in Mfold

HIV-1 RNA ^a^	ΔG (kcal/mol)
NL4-3	-376.6
NL-913	-376.5
NL-1907	-378.2
NLΔSL1	-363.1
NLΔSL1-913	-363.0
NLΔSL1-1907	-364.6

^a ^Genomic RNA nt 456 to 2080 of NL4-3, NL-913 and NL-1907, and nt 456 to 2037 of NLΔSL1, NLΔSL1-913 and NLΔSL1-1907 were used for folding predictions.

### Compensatory mutations in Gag partially rescue the SL1 deletion mutant RNA packaging defect

SL1 is not located within a promoter and does not code for any viral protein; thus SL1 deletion did not affect the expression or processing of HIV-1 proteins (Figure 3B) [11,35,37]. Based on previous studies, we predicted that the SL1 deletion reduces packaging efficiency [10,11,33-35,37,40,45] and packaging selectivity of viral RNAs [11,14,34,40,45]. To explore this possibility, quantitative PCR (qPCR) using primer/probe sets specific for HIV-1 genomic RNA, *env *mRNA, or *rev *mRNA were used to measure the amounts of different RNA species packaged into the virion. The amount of HIV-1 genomic RNA, *env *mRNA or *rev *mRNA in the virion is an indication of the packaging efficiency of full-length unspliced, singly spliced, and fully spliced RNA, respectively.

**Figure 5:**
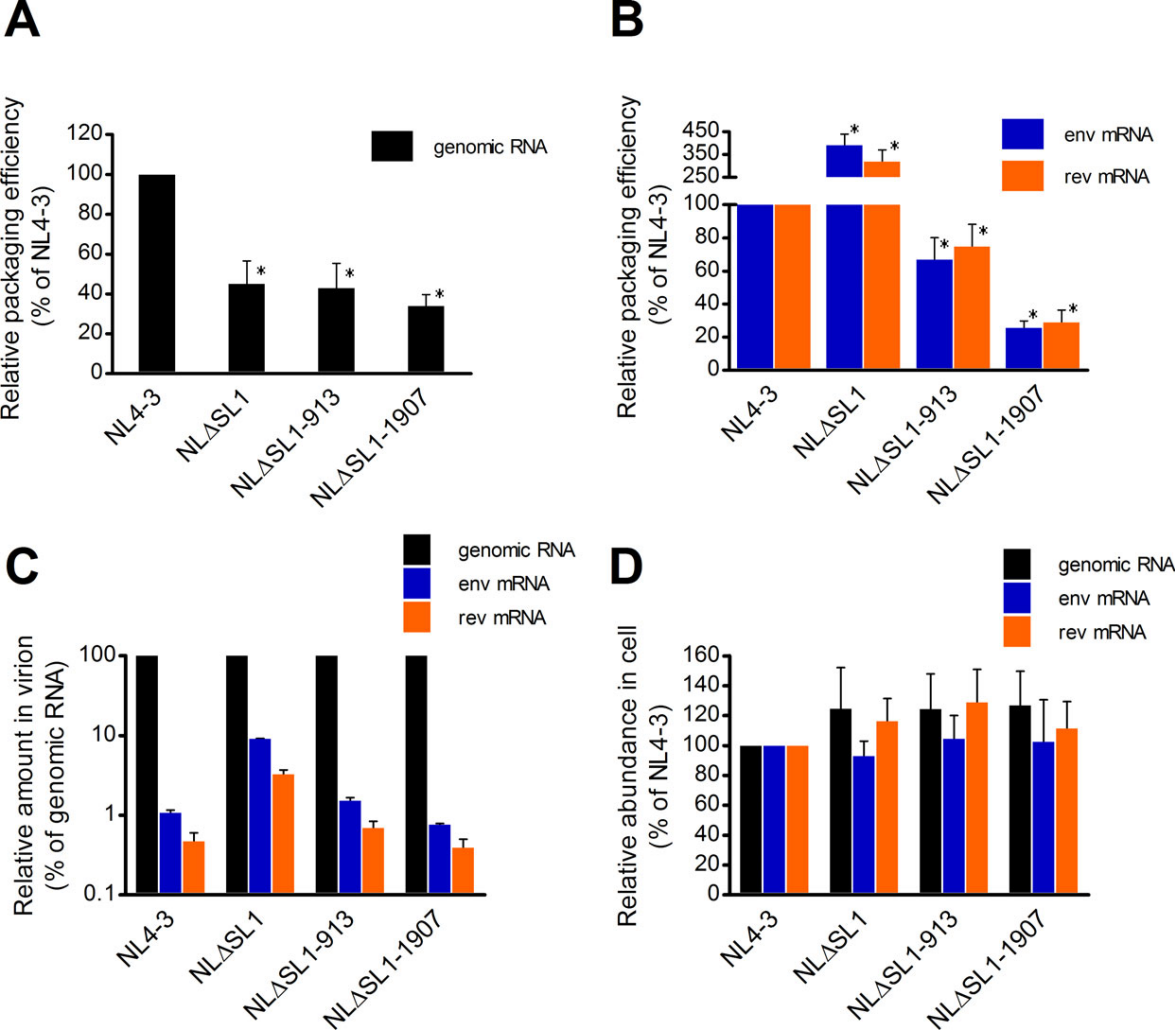
**Quantification of HIV-1 RNA content in the virion**. (A) Efficiency of HIV-1 genomic RNA packaging. RNA was isolated from equivalent amounts of p24 from NL4-3, NLΔSL1, NLΔSL1-913 and NLΔSL1-1907, reverse-transcribed and measured by qPCR with a primer/probe set specific to the HIV-1 unspliced genomic RNA. The amount of NL4-3 genomic RNA was set at 100%. Copy numbers ranged from 2.0 × 10^6 ^to 2.9 × 10^6 ^in four independent experiments. *, indicates *p *< 10^-4 ^and significant deviation from the wild-type copy number as determined by Student's *t *test. (B) Efficiency of spliced HIV-1 RNA packaging. cDNA was subjected to qPCR targeting the env mRNA or rev mRNA sequence as described above. The amount of NL4-3 spliced mRNA was set at 100%. Copy numbers of env mRNA ranged from 24,042 to 28,865, and rev mRNA from 8,387 to 14,335 in four independent experiments. *, indicates significant deviation from the wild-type copy number as determined by Student's *t *test; *p *< 10^-4^, except for NLΔSL1-913, *p *< 10^-3^. (C) Relative amounts of HIV-1 genomic RNA and mRNA in the virion. The copy numbers of HIV-1 genomic RNA and env and rev mRNA were used to calculate the relative amount of mRNA in the virion [(mRNA copy/genomic RNA copy) × 100] and normalized to genomic RNA level. (D) Determination of viral RNA expression in producer cells. Total RNA was isolated from 293T cells transfected with the corresponding vectors and reverse transcribed. The cDNA was quantified by qPCR with primer/probe sets specific for the HIV-1 genomic RNA, *env *mRNA and *rev *mRNA sequences. The copy number in each sample was adjusted for input by the level of PBGD mRNA and for transfection efficiency by GFP expression from a co-transfected reporter construct. The amount of NL4-3 RNA was set at 100%.

We found that the genomic RNA of NLΔSL1 was packaged about half as efficiently as that of NL4-3 (Figure 5A). This result supports the notion that SL1 plays a role in binding Gag during packaging [7,11,46]. In contrast, 3- to 4-fold more NLΔSL1 *env *and *rev *mRNA was packaged into the virion compared to the wild type (Figure 5B) consistent with previous studies showing that SL1 deletion led to an increased packaging of spliced viral RNAs into the virion [11,14,34,40,45]. The deletion in SL1 increased the amount of spliced mRNA over the amount of genomic RNA by 7- to 9-fold (Figure 5C). The abnormal amount of spliced and unspliced NLΔSL1 RNA in the virions was not due to differences in expression, as the RNAs of NL4-3 and NLΔSL1 showed similar expression levels in the producer cells (Figure 5D).

It is possible that the packaging of excess spliced viral mRNA in NLΔSL1 mutants is associated with the reduced production of infectious virions, thereby reducing the overall infectivity of the virus. In support of this hypothesis, we found that the compensatory mutations did not rescue the defect in packaging HIV-1 genomic RNA (Figure 5A), but rather that both the NLΔSL1-913 and NLΔSL1-1907 revertants efficiently excluded spliced viral mRNA from packaging (Figure 5B). The SL1 deletion mutant carrying the mutation in MA, NLΔSL1-913, had about 1.5-fold less *env *and *rev *mRNA in the virion compared to the wild type, whereas the SP1 mutant, NLΔSL1-1907, had about a 4-fold reduction in viral mRNA species in the virion. In addition, both mutations restored the relative amount of spliced mRNA and unspliced genomic RNA in the virion similar to that of the wild type (Figure 5C). These results are consistent with a previous study demonstrating that HIV-1 of the BH10 strain acquires mutations in the MA (V35I) and SP1 (T12I) domains to compensate for the SL1 deletion [34]. In that study, the lone SP1 mutation was sufficient to restore the packaging efficiency and specificity of the Gag, however, SL1 deletion revertant carrying only the MA mutation was not characterized. Moreover, our study supports the notion that the SP1 domain may have a role in HIV-1 RNA packaging [47].

Previous studies have shown that SL1 deletion impairs plus-strand HIV-1 DNA transfer in RT [37,39]. In addition, recombination is restricted in a 2-kb region immediately downstream of SL1 mutations [48] affecting the efficiency of RT and the synthesis of full-length HIV-1 DNA [49]. However, it is unlikely that the mutations in MA and SP1 restore infectivity by rescuing the defects in RT. Indeed, NLΔSL1-913 and NLΔSL1-1907 are still defective in plus-strand DNA transfer (Ristic and Chin, unpublished data) indicating that the compensatory mutations do not have a role in RT. On the other hand, because HIV-1 spliced mRNA does not contain the *gag *sequence [44], the two compensatory mutations are unlikely to affect the packaging efficiency of spliced mRNA at the RNA level. Therefore, the mutations in MA and SP1 likely enable the Gag polyprotein to effectively exclude spliced NLΔSL1 mRNA during packaging. The compensatory mutations led to changes in part of the predicted secondary structures of the HIV-1 genomic RNA, but the SLs remained unchanged (Additional file 2: Figure S2). However, despite the changes in predicted secondary structure, the packaging efficiency of HIV-1 genomic RNA was not altered, suggesting that the SLs are the dominating *cis*-acting element in the packaging process. Further experiments studying viral RNA packaging efficiency by supplying the mutant Gag *in trans *are needed to confirm this observation. In addition, fluorescence microscopy analysis on the mutant Gag within the cell may be necessary to exclude the possibility that the mutations have changed the subcellular localization or trafficking of Gag, resulting in a change in RNA binding preference.

### Reduction of HIV-1 genomic RNA is accompanied by an increase in packaging of cellular RNA into the SL1 deletion mutant virion

HIV-1 packages cellular RNA into the virion [40,50-54]. A previous study has shown that in the absence of packaging signal, murine leukemia virus and HIV-1 package less genomic RNA and more cellular mRNA, but maintain roughly the same amount of RNA as the wild-type virion [50]. In this study, the absence of SL1 led to a reduction of HIV-1 genomic RNA in the virion (Figure 5A). It is possible that the genomic RNA in the SL1 deletion virion was replaced by host RNA and that the virion maintained an RNA level similar to that of wild type. To characterize the cellular RNA packaged into the wild-type and SL1 deletion mutant virions, we used qPCR to measure the packaging efficiency of Y1, Y3, and signal recognition particle (SRP) RNAs, which are the most abundant cellular RNAs in the HIV-1 virion [52,53].

**Figure 6:**
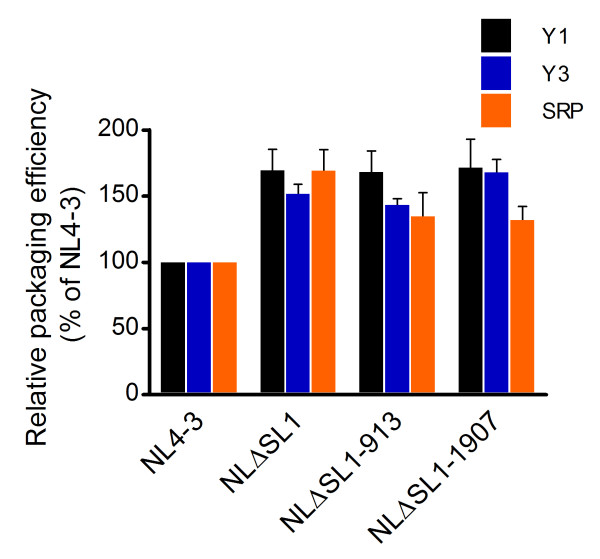
**Characterization of the cellular RNA in the wild-type and SL1 deletion mutant virions**. The same cDNA preparations used to measure HIV-1 RNA content in the virion in Figure 5 were subjected to qPCR characterization targeting cellular Y1, Y3 and SRP RNA. The amount of cellular RNA in the NL4-3 virion was set at 100%. Copy numbers for Y1, Y3 and SRP RNA were 275-362, 1,710-2,006 and 1.1 × 10^6^-1.3 × 10^6^, respectively, as determined in four independent experiments.

We found that in the absence of SL1, Gag packaged about 1.5- to 1.7-fold more Y1, Y3 and SRP RNA into the virion compared to wild type (Figure 6). The revertant Gag did not affect the packaging efficiency of the cellular RNA, suggesting that the increased level of cellular RNA did not affect the infectivity of the virus. Thus, it appears that cellular RNAs were packaged into the virion to fill in the "void" caused by the reduction of genomic RNA in the SL1 deletion mutants. This is consistent with previous studies showing that reduced packaging of genomic RNA is accompanied by increased incorporation of cellular RNA in the virion [40,50]. We compared the RNA copy numbers in the wild-type and NLΔSL1 virions and found that increased copies of *env *and *rev *mRNA, Y1, Y3, and SRP RNA in the NLΔSL1 virion accounted for approximately 67% of the reduction in HIV-1 genomic RNA in the virion. These data suggest that in addition to the viral mRNAs and cellular RNAs reported here, Gag also packages other RNA species to replace the decreased amount of HIV-1 genomic RNA in the NLΔSL1 virion. This also hold true for the revertant virions which likely package other RNA species to replace the decreased amount of HIV-1 genomic and spliced RNA.

### The associations between Gag and HIV-1 RNA correlate with the preference of Gag in packaging different species of RNA

The primary recognition sites for NC are the four SLs in the 5' UTR of the HIV-1 genome [7,11-16]. Biochemical analysis has indicated that short RNAs possessing HIV-1 SL2 or SL3 have the highest affinity for NC, whereas those with SL1 or SL4 have lower affinity for NC [46]. Our data indicate that in the absence of SL1, Gag packaged less HIV-1 genomic RNA, but incorporated significantly more spliced HIV-1 mRNA into the virions confirming and extending previous results on the packaging of spliced viral RNA in SL1 mutants [11,14,34,40,45]. Despite the presence of a packaging signal in the NLΔSL1 genomic RNA, the association between the mutant genomic RNA and Gag may have been reduced, leading to the reduction in packaging efficiency. It is also possible that the deletion of SL1 disrupted an essential secondary RNA structure within the 5' leader on the spliced viral mRNA that is important for Gag to actively select and exclude spliced viral mRNA from packaging. In the Mfold analysis, deletion of SL1 changes the structures within the 5' leader of *env *and *rev *mRNA, but the physiological relevance is not clear (data not shown). We therefore propose that the compensatory mutations in MA or SP1 play a role in making Gag more effective in preventing spliced NLΔSL1 mRNA from being packaged. Based on this prediction, we hypothesized that the compensatory mutations in MA or SP1 reduce the association between Gag and spliced viral mRNA, thereby reducing the likelihood of spliced viral mRNA being packaged into the virion. To test this hypothesis, we quantified Gag and HIV-1 RNA association by immunoprecipitation, followed by qPCR as previously described with modifications [55].

**Figure 7:**
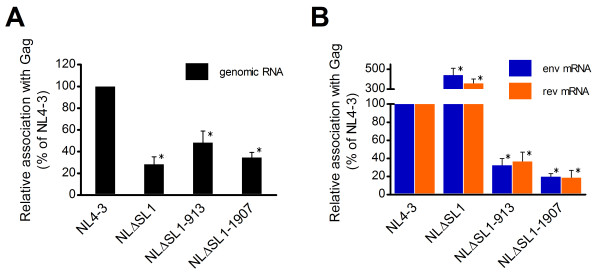
**Characterization of the association between Gag and HIV-1 RNA**. (A) Measurement of the association between Gag and HIV-1 genomic RNA. HIV-1 genomic RNA immunoprecipitated with the Gag was characterized by qPCR using a primer/probe set targeting the unspliced RNA transcript. (B) Measurement of the association between Gag and spliced HIV-1 mRNA. The same cDNA preparation described above was subjected to qPCR using a primer/probe set specific for *env *mRNA and *rev *mRNA sequences. The copy number in each sample was adjusted for input by the cell number and for transfection efficiency by GFP expression from a co-transfected reporter construct. The amount of NL4-3 RNA was set at 100%. Means and SD of three independent experiments are shown. *, indicates *p *< 10^-4 ^and significant deviation from the wild-type copy number as determined by Student's *t *test.

In these experiments, we observed different associations between Gag and the RNAs of NL4-3, the SL1 deletion mutant, and the revertants, although these vectors had similar RNA expression in the producer cells (Figure 4). Specifically, 3-fold less NLΔSL1 genomic RNA was immunoprecipitated by Gag (Figure 7A). Gag carrying mutations in MA or SP1 did not show a significantly altered binding preference and associated with 2- to 3-fold less HIV-1 genomic RNA compared to NL4-3 (Figure 7A). These results suggest that the drop in packaging efficiency of NLΔSL1 genomic RNA is caused by a reduced association of Gag with the ΔSL1 RNA.

We then examined the association between Gag and spliced HIV-1 mRNA. Compared to the wild type, we found that Gag showed an enhanced association with NLΔSL1 spliced mRNA, immunoprecipitating about 4-fold more singly spliced and fully spliced RNA (Figure 7B). This is consistent with the viral RNA packaging result (Figure 5B). Importantly, Gag carrying mutations in MA or SP1 showed significantly reduced association with spliced HIV-1 mRNA compared to the wild type; 3- to 5-fold less HIV-1 mRNA was associated with the revertant Gag compared to that of NL4-3 (Figure 7B). Thus, the association between Gag and viral RNA could directly affect the packaging efficiency of different viral RNA species in the SL1 deletion mutant and its revertants. It will be interesting to find out if the SP1 (T12I) revertant of the SL1-deleted BH10 also use similar mechanism to exclude spliced RNA from encapsidation and restore infectivity [34]. *In vitro *binding assay can be used to confirm the above association between mutant Gag and HIV-1 RNA, but one caveat is that such experiment may not reflect the Gag-RNA association in the physiological condition of a cell. Taken together, these data indicate that HIV-1 can adapt to a severe genetic defect in SL1 through mutations in MA or SP1 that reduce the association of Gag to spliced ΔSL1 HIV-1 RNA, thus effectively preventing these RNAs from being packaged and subsequently increasing the production of infectious virions.

## Conclusion

We demonstrated new pathways for HIV-1 to compensate for a deletion of SL1. A G913A (E42K) mutation in MA and a C1907T (P10L) mutation in SP1 were responsible for the enhanced infectivity of NLΔSL1 in PM-1 cells through partially restoring the packaging specificity of viral RNA. These compensatory mutations may enable Gag to exclude spliced viral RNA from packaging and interfere with the production of infectious virus in SL1 deletion mutants. Prior to this study, no mutations at either of these amino acid positions in Gag had been associated with restoring the infectivity of a mutant. We also present evidence that both mutations affect the Gag-HIV-1 RNA association in a cell-based system. This study provides new insights into the functions of the N-terminal MA domain and SP1 and suggests that both regions may have a role in interacting with different spliced viral RNA transcripts.

## Methods

### Plasmid construction, cell culture and virus

The pNL4-3 molecular clone was obtained from the NIH AIDS Reagent Program [56] and was used for the construction of mutant vectors in this study. A 43-nt region encompassing the SL1 of pNL4-3 (nt position 691 to 733 of proviral DNA) was deleted by site directed mutagenesis to generate pNLΔSL1. The G913A substitution was made to the pNL4-3 and the pNLΔSL1 vectors to generate pNL-913 and pNLΔSL1-913, respectively, by the QuikChange II XL Site-Directed Mutagenesis Kit (Agilent). Using similar approach, the C1907T substitution was made to pNL4-3 and pNLΔSL1 to generate pNL-1907 and pNLΔSL1-1907, respectively.

The HIV indicator cell line TZM-bl and human T cell line PM-1 were obtained from the NIH AIDS Reagent Program [57,58]. Human embryonic kidney cell line 293T and TZM-bl cells were cultured in Dulbecco's modified Eagle's medium. PM-1 cells were cultured in Roswell Park Memorial Institute-1640 medium. Medium was supplemented with 10% fetal calf serum, penicillin (50 U/ml), and streptomycin (50 mg/ml).

Viruses were generated from 293T cells by transfection using the standard calcium phosphate method. Forty-eight hours after transfection, the culture supernatant was harvested and passed through a 0.45-μm-pore size filter to remove cellular debris, and centrifuged through a 20% sucrose cushion. The virus pellet was resuspended in PBS and quantified by p24 ELISA (Advanced BioScience Laboratories). The TCID_50 _of the virus was determined by the Reed and Muench method.

### Infection of PM-1 cells and measurement of viral replication

A total of 5 × 10^5 ^cells were inoculated with 10 ng of p24-normalized virus for 4 hours. Unbound viruses were removed by washing with PBS, and the infected cells were cultured in 6-well plates. Cells were split 1:2 every 7 days. Culture supernatants were collected at different times for detection of infectious virus by TZM-bl cells or measurement of p24 by ELISA.

### Sequencing of the HIV-1 genome

Viral RNA was isolated from the infected culture supernatants using the QIAamp Viral RNA Mini Kit (Qiagen) and converted to cDNA with random hexamers using SuperScript III reverse transcriptase (Invitrogen). The cDNA was amplified using the FastStart High Fidelity PCR System (Roche) in four overlapping fragments covering the near full-length genome of NL4-3. The PCR products were sequenced with overlapping primers, and the resulting sequence contigs were assembled with the Staden Package (PCR and sequencing primer sequences are available upon request) [59]. Every nucleotide was identified by at least two sequence contigs to ensure the accuracy of the DNA sequence.

### Western blot analysis of viral proteins

HIV-1 virion equivalent to 100 ng of p24 was pelleted by centrifugation and resuspended in sample buffer containing 5 mM β-mercaptoethanol. Samples were separated by SDS-PAGE and transferred to PVDF membrane. Blot was probed first with antiserum to HIV-1 p24 or gp120 (obtained from Dr. Michael Phelan through the NIH AIDS Reagent Program) [60] and then with horseradish peroxidase-conjugated secondary antibody (Thermo Scientific). The blot was developed by an enhanced chemiluminescence detection reagent (GE Healthcare).

### Splice site analysis

Total RNA was isolated from 2 × 10^6 ^293T cells transfected with different HIV-1 constructs using TRIzol LS Reagent (Invitrogen). The RNA was converted to cDNA and amplified in a standard PCR using forward primer specific for the NL4-3 U5 (nt 551-570) and reverse primer specific for the vpu (nt 6220-6199). The PCR products were analyzed in a 2% agarose gel, gel purified and cloned into the pCR4-TOPO TA cloning vector (Invitrogen) for sequencing.

### Northern blot analysis of virion RNA dimers

Virion equivalent to 200 ng of p24 was pelleted, and the viral RNA was extracted using TRIzol LS Reagent (Invitrogen) and treated with DNase I. The RNA was separated on a nondenaturing agarose gel in 1× TBE buffer. After electrophoresis, the gel was incubated in 6% formaldehyde at 65°C for 30 min, and the RNA was transferred to a nylon membrane. RNA was cross-linked to the membrane and detected by a 235-nt RNA probe synthesized using the DIG Northern Starter Kit (Roche), which corresponds to the R-PBS region of HIV-1 (456 to 690, NL4-3 numbering). Hybridization and detection of the DIG-labeled RNA probe followed the manufacturer's protocol, which utilized a chemiluminescence detection reagent. RNA on the membrane was quantified by densitometry using ImageJ software.

### RNA secondary structure prediction

RNA secondary structure prediction was performed using Mfold v3.2 [61,62], hosted by the Rensselaer Polytechnic Institute http://mfold.bioinfo.rpi.edu. Folding conditions were 37°C and 1 M NaCl. Sequences comprising nt 456 to 2080 of the NL4-3, NL-913 and NL-1907 genomic RNAs and nt 456 to 2037 of the NLΔSL1, NLΔSL1-913 and NLΔSL1-1907 genomic RNAs were used for the folding predictions.

### Quantitative PCR measurement of RNA

Equivalent amounts of p24 from NL4-3, NLΔSL1, NLΔSL1-913 and NLΔSL1-1907 were treated with DNase I and digested with proteinase K. Viral RNA was isolated with 6 M guanidinium isothiocyanate in the presence of GlycoBlue Coprecipitant (Ambion) and precipitated with isopropanol. The resulting viral RNA was converted to cDNA with random hexamers using SuperScript III reverse transcriptase (Invitrogen) and treated with Dpn I. The cDNA was then subjected to qPCR using primer/probe sets specific for the HIV-1 genomic RNA, *env *mRNA or *rev *mRNA using TaqMan Gene Expression Master Mix (Applied Biosystems) according to the manufacturer's protocol. The same cDNA preparations were also subjected to qPCR using primers specific to the cellular RNA, Y1, Y3 and SRP RNA and the Fast SYBR Green Master Mix (Applied Biosystems). All primer and probe sequences are available upon request.

For the analysis of viral RNA expression in the producer cells, 293T cells transfected with the corresponding vectors were harvested and washed with PBS. Total RNA was isolated from 2 × 10^6 ^cells using TRIzol LS Reagent. The isolated RNA was treated with DNase I before conversion to cDNA using random hexamers. The resulting cDNA was further treated with Dpn I and quantified by qPCR with primer/probe sets specific for the HIV-1 genomic RNA, *env *mRNA and *rev *mRNA sequences as described previously. The transfection efficiency was determined by measuring the percentage of GFP^+ ^expression from a co-transfected reporter construct. The copy number in each sample was normalized to the level of PBGD mRNA.

### Characterization of Gag and HIV-1 RNA association *in vivo*

A previously described protocol with modifications was used [55]. 293T cells transfected with NL4-3, NLΔSL1, NLΔSL1-913 or NLΔSL1-1907 were trypsinized and washed three times with PBS to wash away virion on the cell surface. The cells were suspended in PBS containing 1% formaldehyde and incubated for 10 min at room temperature to cross-link proteins and RNAs in the cell. The cross-liking reaction was quenched with 125 mM glycine for 5 min at room temperature and washed three times with ice cold PBS. Cells were lysed in RIPA buffer (Pierce) and sonicated in the presence of complete protease inhibitor cocktail (Roche) and RNaseOUT (Invitrogen). The cell lysate was clarified by centrifugation and the Gag-RNA cross-linked complex was immunoprecipitated with anti-p24 monoclonal antibody (clone 24-4) (Santa Cruz Biotechnology) bound to Dynabeads Protein G (Invitrogen). The immunoprecipitated complex was washed according to the manufacturer's protocol with the addition of 1 M urea. The sample was then heated to 70°C to reverse the cross-linkages between RNA and Gag. The released RNA was precipitated with isopropanol, digested with DNase I and then subjected to reverse transcription. qPCR was used to measure the amount of HIV-1 genomic RNA, *env *mRNA and *rev *mRNA as described previously. The transfection efficiency was determined by measuring the percentage of GFP^+ ^expression from a co-transfected reporter construct. The number of cell in the input material was standardized using TruCount Absolute-Count tube (BD Biosciences) and flow cytometry.

## Competing interests

The authors declare that they have no competing interests.

## Authors' contributions

N.R. designed and performed experiments, analyzed data and wrote the manuscript. M.P.S.C designed and performed experiments, analyzed data, wrote the manuscript and supervised the project.

## Supplementary Material

Additional file 1 **Supplemental Figure S1**. Replication of NL4-3 and NLΔSL1 in PM-1 cells as determined by p24 ELISA. PM-1 cells were infected with p24-normalized NL4-3 or NLΔSL1. Culture supernatants from the infected PM-1 were collected at different times, and p24 levels were measured by ELISA. Click here for file

Additional file 2 **Supplemental Figure S2**. Predicted secondary structures of NL4-3 and SL1 deletion mutants. Genomic RNA of (A) NL4-3, (B) NL-913 and (C) NL-1907 (nt 456 to 2080) and (D) NLΔSL1, (E) NLΔSL1-913 and (F) NLΔSL1-1907 (nt 456 to 2037) were subjected to Mfold analysis. The SL1 and SL2 and the positions of the MA (913) and SP1 (1907) substitutions are labeled. Click here for file
