# Supplementary figures and images for: Mutations in matrix and SP1 repair the packaging specificity of a Human Immunodeficiency Virus Type 1 mutant by reducing the association of Gag with spliced viral RNA
Source: Retrovirology. 2010 Sep 8;7:73. doi: 10.1186/1742-4690-7-73 (PMC2941742; doi:10.1186/1742-4690-7-73)

## Slide 1
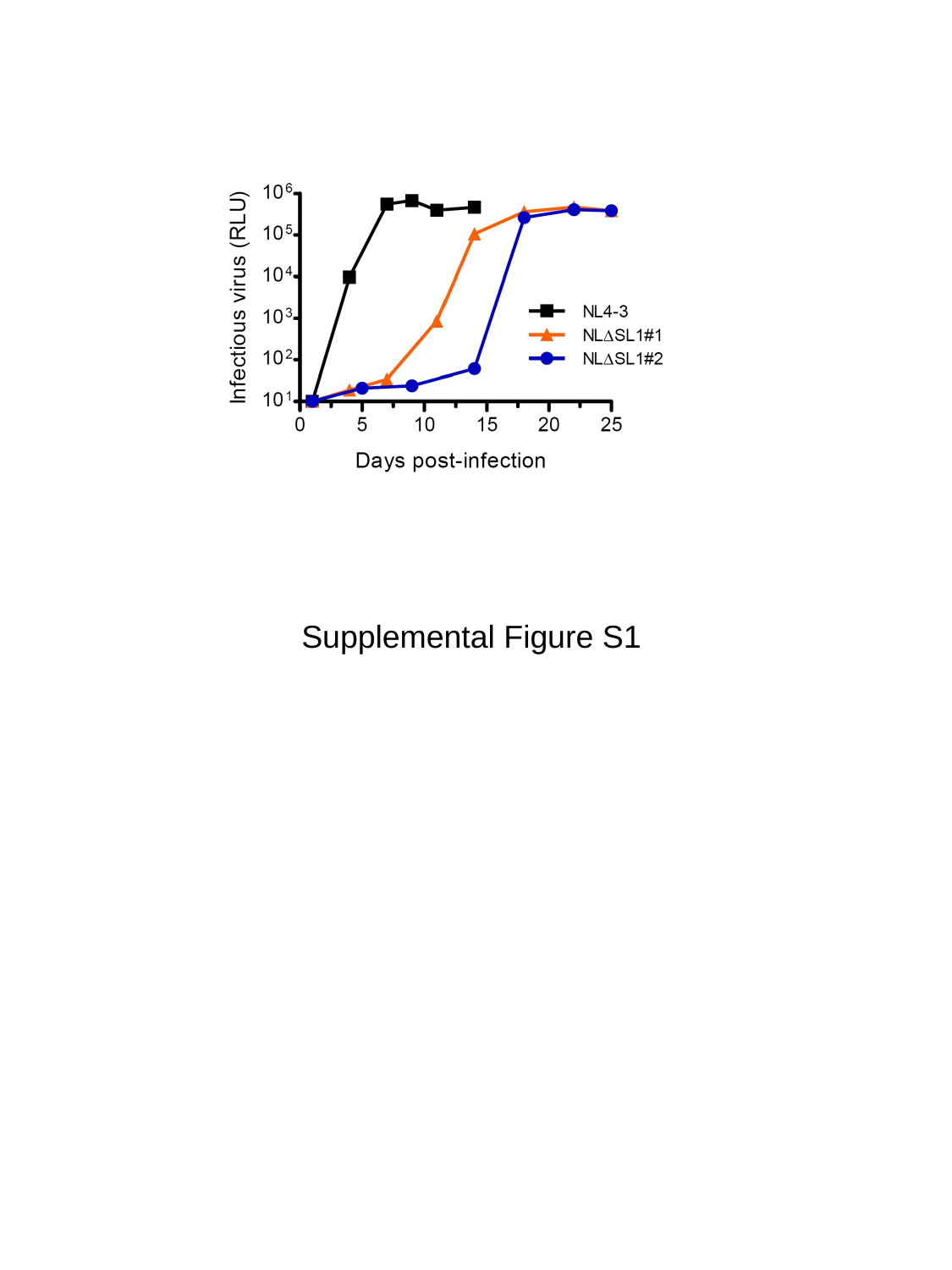

Supplemental Figure S1

Supplement: Additional file 1 — Supplemental Figure S1. Replication of NL4-3 and NLΔSL1 in PM-1 cells as determined by p24 ELISA. PM-1 cells were infected with p24-normalized NL4-3 or NLΔSL1. Culture supernatants from the infected PM-1 were collected at different times, and p24 levels were measured by ELISA. [file 1742-4690-7-73-S1.PPT]

## Slide 1
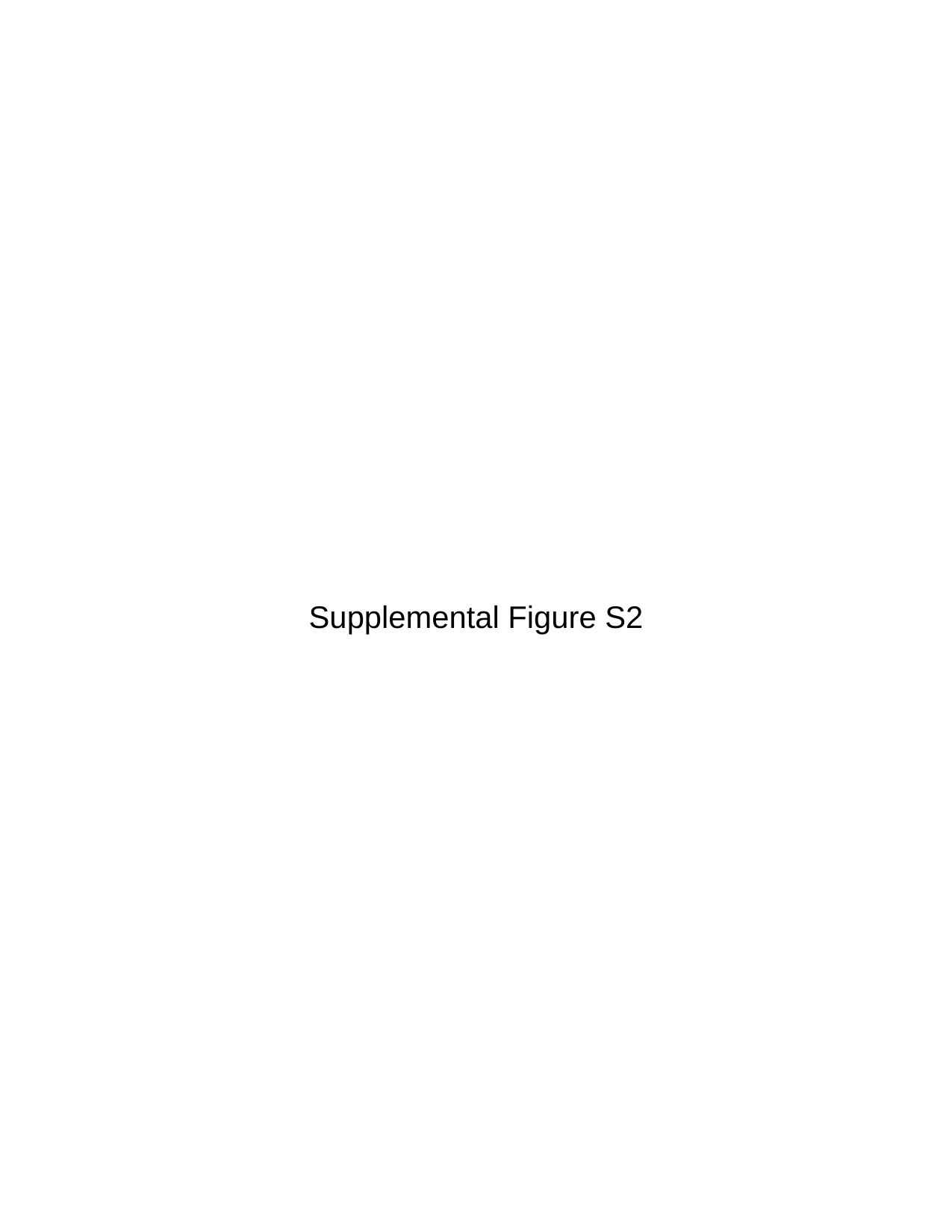

Supplemental Figure S2

## Slide 2
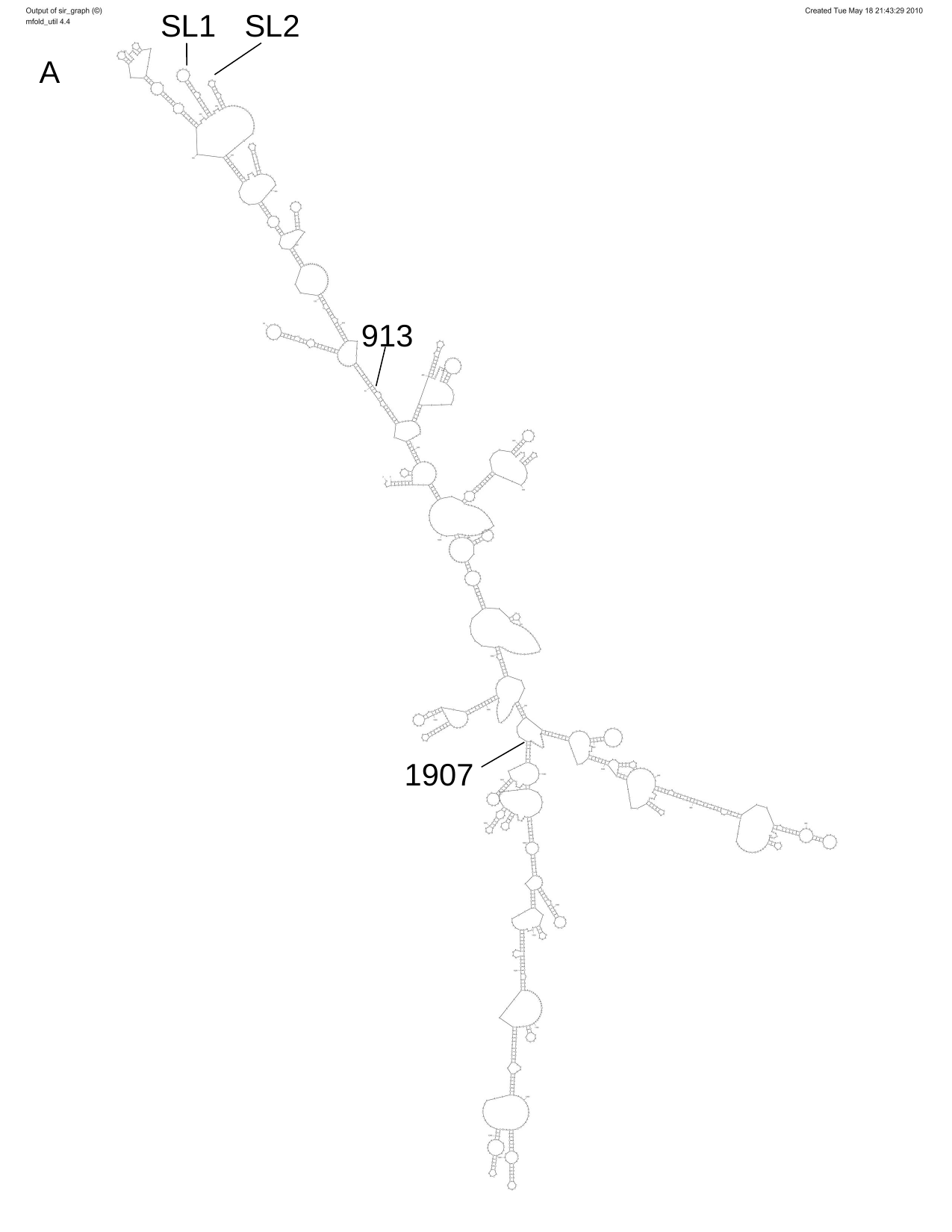

SL1
SL2
A
913
1907

## Slide 3
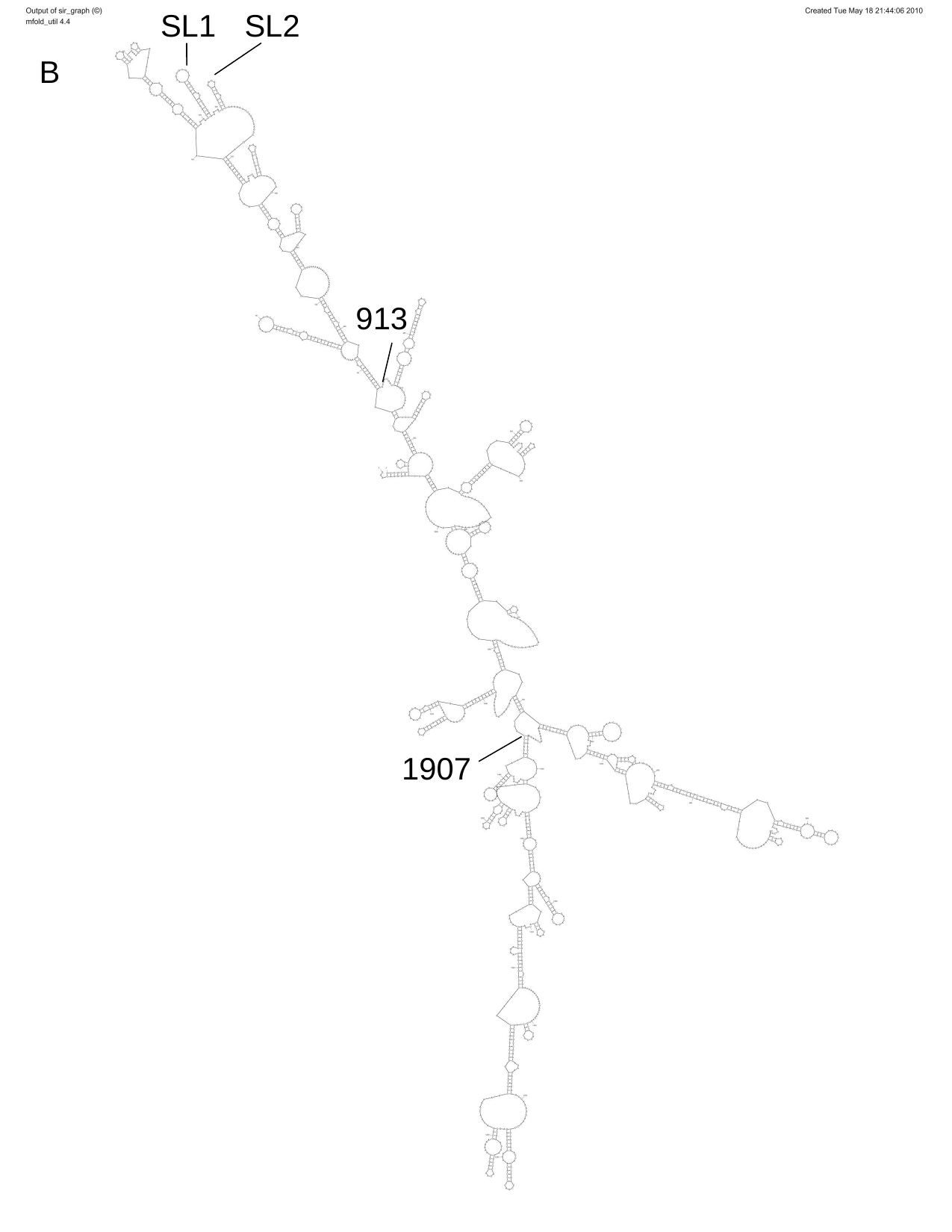

SL1
SL2
B
913
1907

## Slide 4
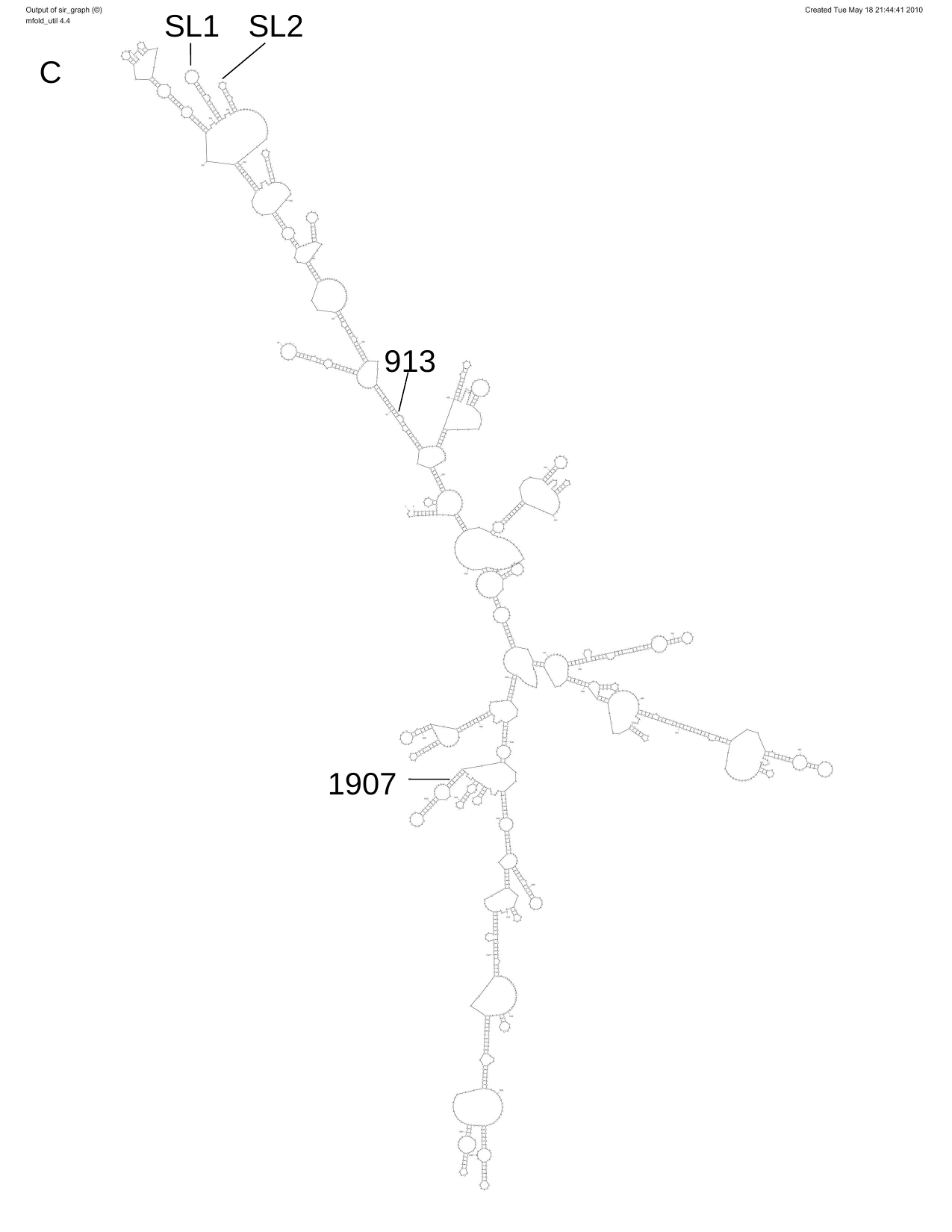

SL1
SL2
C
913
1907

## Slide 5
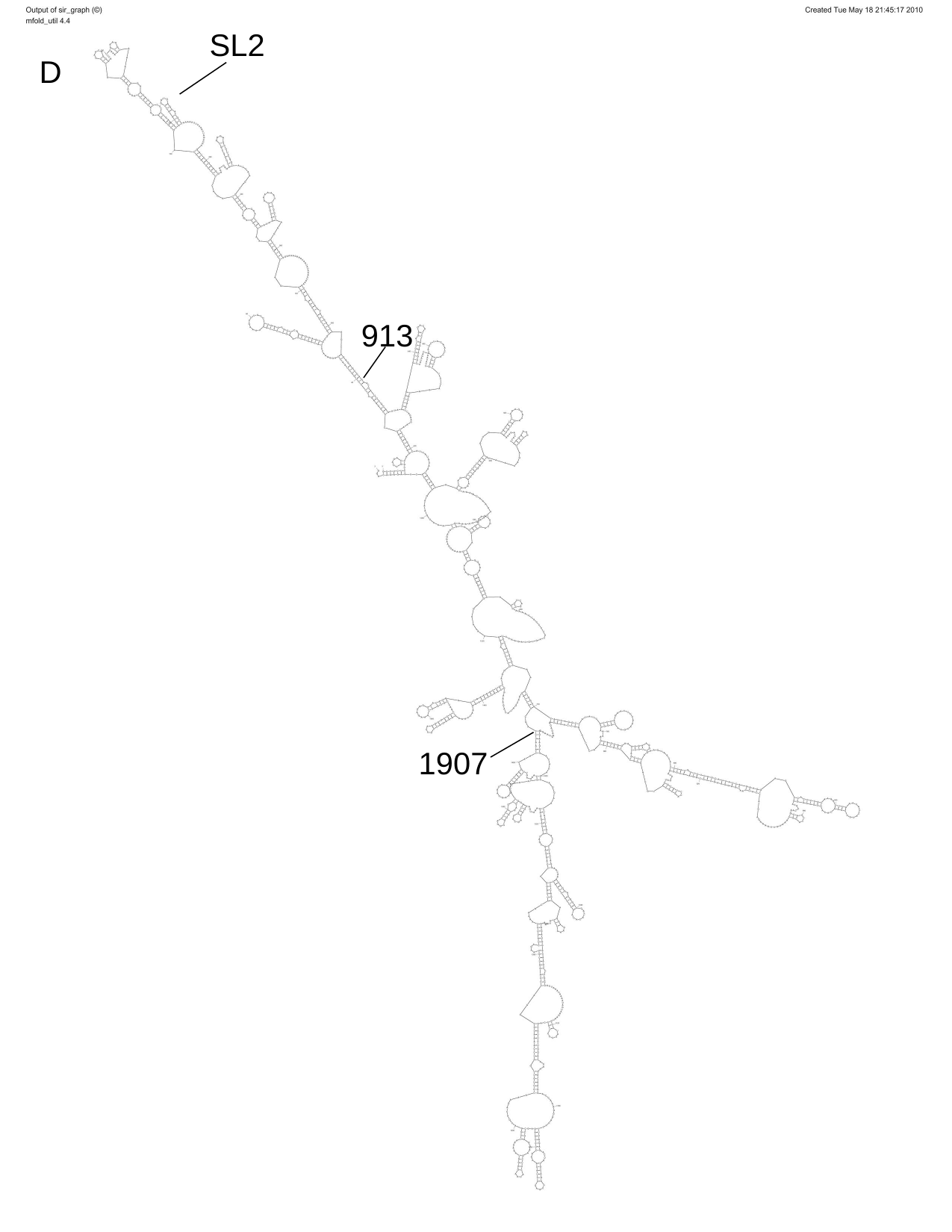

SL2
D
913
1907

## Slide 6
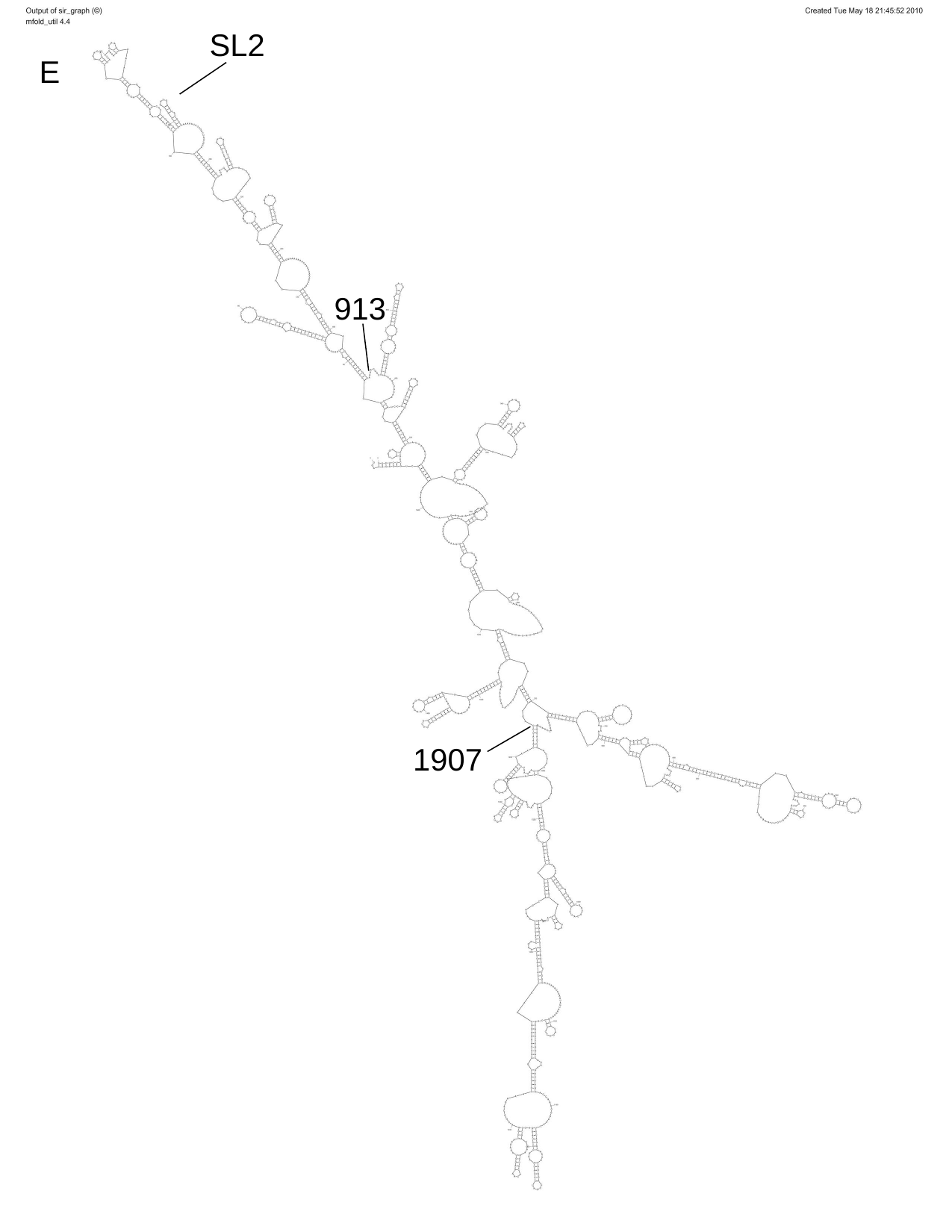

SL2
E
913
1907

## Slide 7
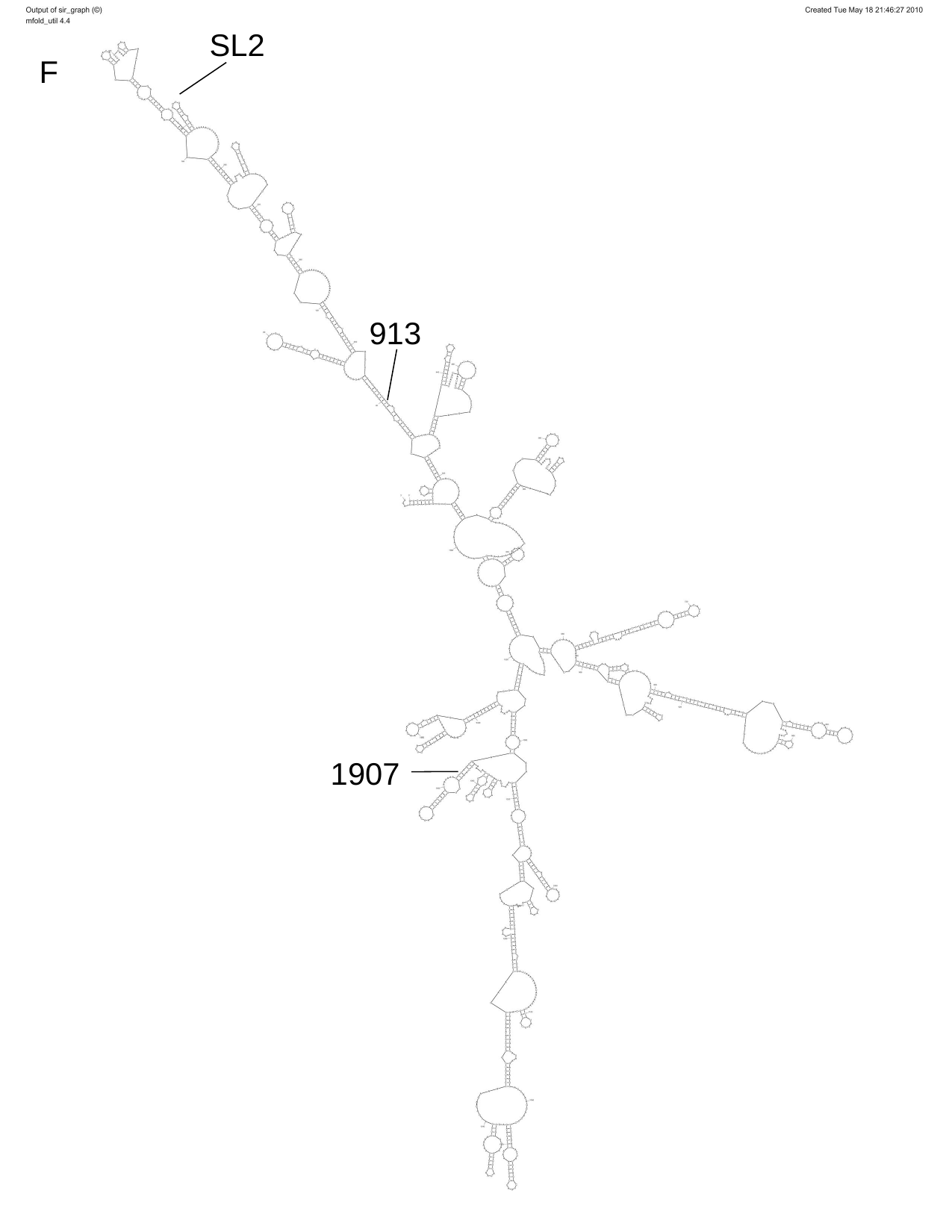

SL2
F
913
1907

Supplement: Additional file 2 — Supplemental Figure S2. Predicted secondary structures of NL4-3 and SL1 deletion mutants. Genomic RNA of (A) NL4-3, (B) NL-913 and (C) NL-1907 (nt 456 to 2080) and (D) NLΔSL1, (E) NLΔSL1-913 and (F) NLΔSL1-1907 (nt 456 to 2037) were subjected to Mfold analysis. The SL1 and SL2 and the positions of the MA (913) and SP1 (1907) substitutions are labeled. [file 1742-4690-7-73-S2.PPT]
